# Association of *Previne Brasil* Program with prenatal care and maternal-child mortality

**DOI:** 10.11606/s1518-8787.2025059006735

**Published:** 2025-10-24

**Authors:** Caio Vieira de Barros Arato, Luciane Miranda Guerra, Livia Fernandes Probst, Antonio Carlos Pereira

**Affiliations:** 1 Universidade Estadual de Campinas. Faculdade de Odontologia de Piracicaba. Departamento de Saúde Coletiva, Odontopediatria e Ortodontia. Piracicaba, SP, Brasil

**Keywords:** Infant Mortality, Maternal Mortality, Socioeconomic Factors, Primary Healthcare

## Abstract

**OBJECTIVE:**

To investigate the impact of prenatal care on the reduction of maternal-child mortality in Brazilian municipalities following the new primary health care financing model, the *Previne Brasil* program.

**METHODS:**

This study comprised a nationwide cross-sectional observational analysis, utilizing secondary data from *Departamento de Informática do Sistema Único de Saúde* (Datasus – Information Technology Department of the Unified Health System), *Sistema de Informação sobre Mortalidade* (SIM – Mortality Information System), *Sistema de Informação sobre Nascidos Vivos* (Sinasc – Live Birth Information System), *Sistema de Informação em Saúde para a Atenção Básica* (Sisab – Primary Health Care Information System), and *Instituto Brasileiro de Geografia e Estatística* (IBGE – Brazilian Institute of Geography and Statistics). Medians of maternal mortality, infant mortality, and prenatal care rates were calculated for Brazilian municipalities from 2016 to 2022. Logistic regression analyses were conducted to assess associations between independent variables (primary care coverage, population size, Gross Domestic Product, and Gini index) and outcomes (variations in maternal and infant mortality rates). Both crude and adjusted odds ratios were estimated, with a significance level of 5%.

**RESULTS:**

Prenatal care rates increased by 86.7%, while maternal mortality rates decreased by 30.9%, with no association between them. Region, primary care coverage, municipal Gross Domestic Product, and population size were associated with variations in maternal mortality rates. The Southern region had a higher likelihood of reducing maternal mortality. No association was found between increased prenatal care rates and reduced infant mortality. Regional location, primary care coverage, population size, and the Gini index were associated with variations in infant mortality rates, with greater reductions observed in more populous municipalities and in the Northeast, Southeast, and Midwest.

**CONCLUSION:**

The *Previne Brasil* program led to an increase in prenatal care consultations in Brazilian municipalities but did not significantly impact the reduction of maternal-child mortality.

## INTRODUCTION

Maternal mortality (MM) and infant mortality (IM) remain significant public health challenges^1,2^, despite being preventable with adequate prenatal care. These indicators are traditionally considered crucial measures of a population's living standards and social well-being. MM is defined as the annual number of deaths among women due to pregnancy, childbirth, or postpartum complications, per 100,000 live births^3^. IM encompasses the probability of a child dying within the first year of life per 1,000 live births, including neonatal mortality (0 to 27 days of life) and post-neonatal mortality (28 days to one year of age), both expressed per 1,000 live births^4^.

Despite coordinated global efforts led by the World Health Organization and the United Nations, reductions in MM and IM have been uneven, with vulnerable groups experiencing less significant improvements. Countries with higher poverty rates and limited access to quality healthcare, particularly in rural or marginalized areas, experience less significant decreases in mortality compared to wealthier regions or countries with better healthcare systems^5^. In Brazil, despite public initiatives and policies implemented in recent decades, the effectiveness of quantitative approaches—focused on measurable data and performance indicators— as well as performance-based payment systems, in which provider compensation is linked to achieving specific targets, has been limited^6^.

In 2004, Brazil launched the National Pact for the Reduction of Maternal and Neonatal Mortality, based on human rights, gender equity, ethnic aspects, and social inequalities^7^. Although progress has been made in reducing IM rates, neonatal mortality remains a persistent concern. Regarding MM, there have been improvements, but the reduction goal set for 2015 was not fully achieved^8,9^.

Despite observed reductions in MM, outcomes are not entirely optimistic. Between 1990 and 2010, there was a decrease from 141 to 68 deaths per 100,000 live births. However, the target of a 75% reduction by 2015 was not achieved. The MM ratio decreased by 8.4% between 2017 and 2018, dropping from 64.5 to 59.1 deaths per 100,000 live births—an improvement, but challenges persist. Recent studies have highlighted the relationship between MM and IM rates and contextual variables, such as demographic and sociocultural aspects^10^. In the United States, effective interventions in primary health care (PHC) have been associated with significant reductions in IM. However, few studies have addressed how political and economic contexts influence health outcomes, considering that developing countries may struggle to define equitable policies to implement effective person-centered healthcare measures^11^.

In Brazil, to address high MM and IM rates, policies have been implemented to reform the funding of PHC within the Brazilian Unified Health System (SUS). The *Previne Brasil* (PB – Prevent Brazil) program, established by Ordinance No. 2,979 on November 12, 2019, is guided by performance indicator No. 1: the "Proportion of pregnant women with at least six prenatal visits, with the first visit by the 12^th^ week of pregnancy." This indicator sets a goal for municipalities to increase the number of prenatal visits by 45%, adopting a predominantly quantitative approach that overlooks the quality of care and other contextual factors that may directly influence mortality outcomes. The program aims to reduce MM and MI rates by rewarding municipalities that meet the parameters through quarterly transfers^12^.

Thus, this study aims to analyze the impact and practical implications of a quantitative approach, focusing on prenatal care rates within PHC and their role in reducing MM and IM rates in Brazilian municipalities, especially after the implementation of PB. By focusing on these results, the study seeks to address gaps in the current understanding of how healthcare financing policies based on service quantity effectively impact health outcomes. The post-implementation period of PB provides a window to assess how changes in PHC funding influence prenatal care practices and, consequently, affects the reduction of mortality rates in Brazil.

## METHODS

### Study Design

This was a nationwide ecological epidemiological study based on secondary data and reported in accordance with the Strengthening the Reporting of Observational studies in Epidemiology (STROBE) guidelines.

### Ethical Aspects

This study was reviewed by the Research Ethics Committee (CEP) of the Piracicaba Dental School at the Universidade Estadual de Campinas (FOP/UNICAMP), in accordance with Resolution No. 466/12 of December 12, 2012, from the National Health Council, and its complementary resolutions (240/97, 251/97, 292/99, 303/2000, 304/2000, 340/2004, 346/2005, and 441/2011). However, since only secondary data from public databases were used, this study was exempt from CEP evaluation, as per CEP Official Letter 05/2023.

### Outcome Measurement and Data Source

Individual spreadsheets and databases queries were initially created on the websites of the responsible organizations (described below) for each variable under study. After data collection, the information was transferred to an Excel spreadsheet using the VLOOKUP command for database management. For municipalities lacking data provided by the aforementioned responsible organizations, a period (.) was assigned to their corresponding cell—a method employed by the SAS program to generate the analysis.

The following are definitions and categorizations of the variables studied:

MM rate: number of deaths of women due to issues related to pregnancy, childbirth, and postpartum, divided by the number of live births to resident mothers, multiplied by 1,000. Data were provided by *Departamento de Informação e Informática do Sistema Único de Saúde* (Datasus – Department of Information Technology of the Brazilian SUS)^13^.

IM rate: number of children who died between birth and one year of age per 1,000 live births. Data were obtained from the *Sistema de Informação sobre Mortalidade* (SIM – Mortality Information System), a national epidemiological surveillance system that collects data on deaths to provide mortality information, and the *Sistema de Informação sobre Nascidos Vivos* (Sinasc – Live Birth Information System), which collects national birth data^13^.

Prenatal care rate: gross total of prenatal care in the municipality over the number of pregnant women in the municipality. Data were obtained from the *Sistema de Informação em Saúde para a Atenção Básica* (Sisab – Primary Health Care Information System), a Brazilian Ministry of Health platform used to monitor population health^14^.

PHC coverage: proportion of the population covered by primary health care teams. Data were obtained from Sisab^14^.

Municipal Gross Domestic Product (GDP): raw data from *Instituto Brasileiro de Geografia e Estatística* (IBGE – Brazilian Institute of Geography and Statistics), the official institute responsible for statistical data collection and analysis^15^.

Population: total population number according to census and projections provided by IBGE^15^.

Gini index: measure of income inequality, ranging from 0 (perfect equality) to 1 (maximum inequality). Raw data from IBGE^15^.

After adjustments and data verification in the spreadsheets, datasets were forwarded to the statistician responsible for conducting the analysis and creating the tables.

### Statistical Analysis

Initially, medians of maternal, infant, and prenatal mortality rates were calculated for all Brazilian municipalities during the years before (2016 to 2018) and after (2019 to 2022) the implementation of the new PHC financing model, PB. The median was chosen as a measure of central tendency due to its robustness against outliers. Subsequently, municipalities were categorized based on whether they experienced a reduction in mortality rates after the implementation of the program. Similarly, municipalities were categorized based on whether there was an increase in prenatal care rates post-implementation. Additionally, medians were calculated for PHC coverage, municipal population, municipal GDP, and the Gini index, enabling the categorization of municipalities based on these potential confounding variables. Table 1 summarizes the categorizations of analyzed variables.

**Table 1:** Variables used in the study.

Type	Variable	Categorization
Outcome 1	Variation in MM rate	Decreased / remained the same or increased
Outcome 2	Variation in IM rate	Decreased / remained the same or increased
Independent variable 1	Regions of the country	N / NE / S / SE / CO
Independent variable 2	Variation in prenatal care rate	Remained the same or decreased / increased
Independent variable 3	Basic attention coverage	< 100% / 100% (median)
Independent variable 4	Municipal GDP	≤ 20,159 / > 20,159 (median)
Independent variable 5	Population	≤ 11,584 / > 11,584 (median)
Independent variable 6	Gini index	≤ 0.5 / > 0.5 (median)

N: North; NE: Northeast; S: South; SE: Southeast; CO: Midwest; MM: maternal mortality; IM: infant mortality; GDP: Gross Domestic Product.

Logistic regression analyses were then conducted to examine the association between each independent variable and the outcomes. From these analyses, crude odds ratios (ORs) with 95% confidence intervals (95%CI) were estimated. Variables with p < 0.20 in individual analyses were included in multiple logistic regression models. The final model retained variables with p ≤ 0.05 after adjustments for other variables. Adjusted ORs with 95%CI were estimated from the multiple models. Model fit was assessed using the Akaike Information Criterion (AIC). All analyses were conducted using the R program (R Core Team, 2023), with a significance level of 5%.

## RESULTS

Data from 5,570 Brazilian municipalities were evaluated before and after the implementation of PB. As observed in Table 2, following implementation, 86.7% of municipalities reported an increase in prenatal care rates. However, a decrease in MM occurred in only 30.9% of the municipalities. No statistically significant association was found between the increase in prenatal care rate and the decrease in MM rates (OR = 0.92; 95%CI: 0.78–1.09; p > 0.05). Among the municipalities with increased prenatal care rates, 30.7% had a decrease in MM rates, compared to 32.5% among those without an increase.

**Table 2:** Analyses (adjusted and unadjusted) of associations with maternal mortality rate variations in Brazilian municipalities, following the new financing model of the PB program.

Variables	Category	n (%)	Variation		OR (95%CI)	p-value	OR^a^ (95%CI)	p-value
			Decreased^a^	Remained the same or increased				
			n (%)	n (%)				
Global	-	5,570 (100)	1,721 (30.9)	3,849 (69.1)	-	-	-	-
Variation in prenatal care rate	Remained the same or decreased	738 (13.3)	240 (32.5)	498 (67.5)	Ref		-	-
	Increased	4,805 (86.7)	1,476 (30.7)	3,329 (69.3)	0.92 (0.78–1.09)	0.3242		
Country regions	North	450 (8.1)	121 (26.9)	329 (73.1)	Ref		Ref	
	Northeast	1,794 (32.2)	544 (30.3)	1,250 (69.7)	1.18 (0.94–1.49)	0.1540	1.04 (0.82–1.32)	0.7302
	South	1,191 (21.4)	403 (33.8)	788 (66.2)	1.39 (1.09–1.77)	0.0072	1.46 (1.12–1.89)	0.0047
	Southeast	1,668 (30)	511 (30.6)	1,157 (69.4)	1.20 (0.95–1.52)	0.1235	1.20 (0.95–1.52)	0.1277
	Midwest	467 (8.4)	142 (30.4)	325 (69.6)	1.19 (0.89–1.58)	0.2392	1.23 (0.91–1.66)	0.1783
Basic attention coverage	< 100%	1,640 (29.4)	402 (24.5)	1,238 (75.5)	Ref		Ref	
	100%^b^	3,930 (70.6)	1,319 (33.6)	2,611 (66.4)	1.56 (1.36–1.77)	< 0.0001	1.27 (1.10–1.47)	0.0014
Municipal GDP	≤ 20,159^b^	2,785 (50)	906 (32.5)	1,879 (67.5)	1.16 (1.04–1.31)	0.0083	1.33 (1.14–1.55)	0.0002
	> 20,159	2,785 (50)	815 (29.3)	1,970 (70.7)	Ref		Ref	
Population	≤ 11,584^b^	2,786 (50)	1,018 (36.5)	1,768 (63.5)	1.70 (1.52–1.91)	< 0.0001	1.51 (1.33–1.72)	< 0.0001
	>11,584	2,784 (50)	703 (25.2)	2,081 (74.8)	Ref		Ref	
Gini index	≤ 0.5^b^	2,711 (48.7)	848 (31.3)	1,863 (68.7)	1.04 (0.92–1.16)	0.5389	-	-
	> 0.5	2,854 (51.3)	871 (30.5)	1,983 (69.5)	Ref			

Ref: reference category for independent variables; OR: odds ratio; 95%CI: 95% confidence interval. PHC: primary health care; PB: Previne Brasil Program; AIC: Akaike Information Criterion; GDP: Gross Domestic Product.

a Outcome event.

b Median AIC (empty model) = 6,889.63; AIC (final model) = 6,790.52.

**Figure 1:**
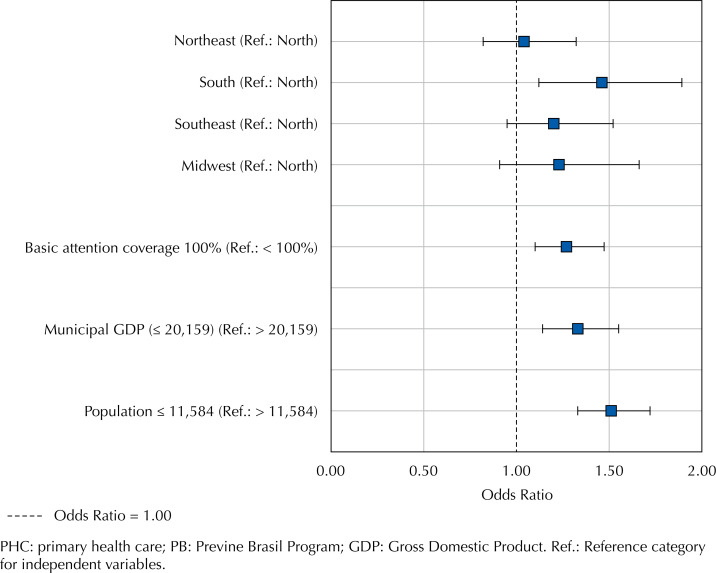
Odds ratios and confidence intervals of associations with the decrease in maternal mortality rate in Brazilian municipalities following the new financing model of the PB program.

PHC: primary health care; PB: Previne Brasil Program; GDP: Gross Domestic Product. Ref.: Reference category for independent variables.

Region (OR = 1.46; 95%CI: 1.12–1.89; p < 0.05), PHC coverage (OR = 1.27; 95%CI: 1.10–1.47; p < 0.05), municipal GDP (OR = 1.33; 95%CI: 1.14–1.55; p < 0.05), and population size (OR = 1.51; 95%CI: 1.33–1.72; p < 0.05) were significantly associated with variations in MM rates. The Southern region exhibited a higher likelihood of decreasing MM rates compared to the Northern region, as depicted in Figure 1. Furthermore, municipalities with 100% PHC coverage, lower GDP (≤ R$ 20,159), and smaller population sizes were more likely to achieve a reduction in MM. Specifically, 33.6% of municipalities with full PHC coverage experienced a decrease in MM, compared to 24.5% among those with lower coverage. Additionally, 36.5% of municipalities smaller population saw a decrease in this rate, compared to 25.2% among those with larger populations.

**Figure 2:**
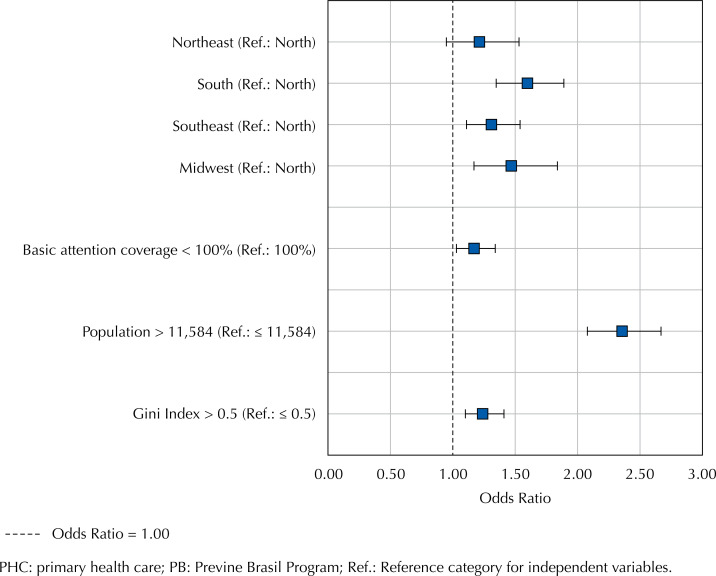
Odds ratios and confidence intervals of associations with the infant mortality rate decrease in Brazilian municipalities following the new financing model of the PB program.

PHC: primary health care; PB: Previne Brasil Program; Ref.: Reference category for independent variables.

Similarly, no statistically significant association was found between the increase in prenatal care rates and the decrease in IM rates (OR = 1.14; 95%CI: 0.97–1.34; p > 0.05), as indicated in Table 3. Overall, 42.6% of municipalities experienced a decrease in IM. Among those with increased prenatal care rates, 43.2% had a decrease in IM, compared to 40.0% among those without an increase. Variations in IM rates were significantly associated with region (OR = 1.60; 95%CI: 1.35–1.89; p < 0.05), PHC coverage (OR = 1.17; 95%CI: 1.03–1.34; p < 0.05), population size (OR = 2.36; 95%CI: 2.08–2.67; p < 0.05), and the Gini index (OR = 1.24; 95%CI: 1.10–1.41; p < 0.05), with a higher likelihood of decrease in more populous municipalities, as illustrated in Figure 2. The Northeast, Southeast, and Midwest regions had higher odds of decreasing IM compared to the Southern region. Among more populous municipalities, 54.8% experienced a decrease in IM, whereas this rate was 30.5% among less populous areas. Furthermore, 50.6% of municipalities with less than full PHC coverage had a decrease in IM, compared to 39.3% among those with 100% coverage.

**Table 3:** Analyses (adjusted and unadjusted) of associations with the infant mortality rate variation in Brazilian municipalities following the new financing model of the PB program.

Variables	Category	n (%)	Variation		OR (95%CI)	p-value	OR^a^ (95%CI)	p-value
			Decreased^a^	Remained the same or increased				
			n (%)	n (%)				
Global	-	5,570 (100)	2,375 (42.6)	3,195 (57.4)	-	-	-	-
Variation in prenatal care rate	Remained the same or decreased	738 (13.3)	295 (40)	443 (60)	Ref		-	-
	Increased	4,805 (86.7)	2,074 (43.2)	2,731 (56.8)	1.14 (0.97–1.34)	0.1030		
Country regions	North	450 (8.1)	203 (45.1)	247 (54.9)	1.71 (1.37–2.13)	< 0.0001	1.21 (0.95–1.53)	0.1266
	Northeast	1,794 (32.2)	894 (49.8)	900 (50.2)	2.06 (1.77–2.40)	< 0.0001	1.60 (1.35–1.89)	< 0.0001
	South	1,191 (21.4)	387 (32.5)	804 (67.5)	Ref		Ref	
	Southeast	1,668 (30)	688 (41.2)	980 (58.8)	1.46 (1.25–1.70)	< 0.0001	1.31 (1.11–1.54)	0.0011
	Midwest	467 (8.4)	203 (43.5)	264 (56.5)	1.60 (1.28–1.99)	< 0.0001	1.47 (1.17–1.84)	0.0010
Basic attention coverage	< 100%	1,640 (29.4)	830 (50.6)	810 (49.4)	1.58 (1.41–1.78)	< 0.0001	1.17 (1.03–1.34)	0.0186
	100%^b^	3,930 (70.6)	1,545 (39.3)	2,385 (60.7)	Ref		Ref	
Municipal GDP	≤ 20,159^b^	2,785 (50)	1,272 (45.7)	1,513 (54.3)	1.28 (1.15–1.43)	< 0.0001	-	-
	> 20,159	2,785 (50)	1,103 (39.6)	1,682 (60.4)	Ref			
Population	≤ 11,584^b^	2,786 (50)	849 (30.5)	1,937 (69.5)	Ref		Ref	
	> 11,584	2,784 (50)	1,526 (54.8)	1,258 (45.2)	2.77 (2.48–3.09)	< 0.0001	2.36 (2.08–2.67)	< 0.0001
Gini index	≤ 0.5^b^	2,711 (48.7)	979 (36.1)	1,732 (63.9)	Ref	< 0.0001	Ref	0.0007
	> 0.5	2,854 (51.3)	1,395 (48.9)	1,459 (51.1)	1.69 (1.52–1.88)		1.24 (1.10–1.41)	

Ref: reference category for independent variables; OR: odds ratio; 95%CI: 95% confidence interval. PHC: primary health care; PB: Previne Brasil Program; AIC: Akaike Information Criterion; GDP: Gross Domestic Product.

a Outcome event.

b Median. AIC (empty model) = 7,562.74; AIC (final model) = 7,183.30.

## DISCUSSION

The implementation of the PB program revealed significant implications for Brazilian municipalities, as evidenced by the findings of this study. While a direct cause-and-effect relationship cannot be established, the data suggest a potential interaction between variables. The program, based on quantitative goals—particularly the proportion of pregnant women with at least six prenatal consultations as its primary performance indicator—was associated with increased prenatal care rates in 86.7% of the analyzed municipalities. However, it is important to consider that other municipal and state-level initiatives, which were not assessed in this study, may have influenced these results. Further research incorporating additional policies and funding strategies is needed. These findings align with Schönholzer's^16^ observations on the increasing trend in indicators following policy implementation. Conversely, other authors, such as Massuda^12^, have raised concerns about performance-based financing in public systems, arguing that while it may lead to quantitative improvements, it may also compromise the quality of care provided.

Despite the observed increase in prenatal care rates, the introduction of the new financing model in 2019 was not associated with significant reductions in MM or IM. The 30.9% decrease in MM in some municipalities showed no significant relationship (p > 0.05) with the increased prenatal consultations proposed by the program. This lack of association aligns with findings from systematic reviews by Witter et al.^17^ and Scott et al.^18^, which suggest that despite the rising trend of using performance-based financial incentives to improve the PHC quality, there is insufficient evidence to support their application for improving the quality of health services.

These findings challenge the assumption that increasing the number of prenatal consultations alone can reduce MM, emphasizing the need to assess structural, contextual, and vulnerability conditions for the development of more effective public policies in mitigating MM. In line with data from this study, analyses of MM in Brazil have showed higher rates among socially vulnerable women, such as low-income and non-White women, highlighting the crucial need for public health policies that account for disparities and situational contexts^19^. This contrasts with policies that overlook differences and variability among Brazilian municipalities for payment based on productive goal-setting.

Furthermore, the findings of this study indicate that the implementation of the program was not associated with a decrease in IM rates in 42.6% of the analyzed municipalities. The lack of association does not suggest that an increase in prenatal consultations alone can positively influence IM outcomes. This aligns with previous research emphasizing the importance of adequate prenatal care in preventing complications and promoting healthier births. These results are consistent with Chuang et al.^20^, who discuss how contextual characteristics, such as socioeconomic conditions, directly impact IM rates in developing countries. These findings suggest that, while a quantitative increase in prenatal consultations is relevant for municipal funding, other factors—such as the quality of care, continuity of postpartum care, access to specialized services, and more equitable income distribution—, are crucial for reducing IM.

The analysis of demographic and socioeconomic characteristics of Brazilian municipalities revealed that in municipalities with smaller populations, lower GDP, and 100% PHC coverage were more likely to experience a reduction in MM rates. This finding suggests that in less complex and more socially cohesive contexts, the effective implementation of policies may yield a greater impact. Nevertheless, it is inferred that aligning these findings with other research suggesting that the organization of the chosen model for PHC, such as the family health strategy (FHS), can directly contribute to the reduction of IM^21^. However, the differentiation of municipalities according to the PHC model was not considered in this study, raising the possibility that municipalities with higher FHS coverage, consistent with Guerra et al.^21^, may have greater chances of reducing mortality.

However, this study emphasizes that in municipalities with larger populations, higher Gini index, and lower PHC coverage, the increased likelihood of decreasing IM rates indicates that the PB impact may vary depending on socioeconomic context. Interestingly, smaller municipalities were more likely to reduce MM rates, whereas larger municipalities were more likely to reduce IM rates. This pattern may be linked to financial incentives and PHC coverage, as previous studies have shown a gradual increase in *per capita* health funding as municipal population size decreases, alongside higher FHS coverage in smaller municipalities^22^. This observation underscores the importance of tailored approaches to address socioeconomic disparities, highlighting the need to consider the diversity of contexts when implementing public health policies. This implies treating municipalities equitably, accounting for their unique characteristics.

Supporting these findings, Oliveira and Wendland^23^ reported a positive association between economic aspects, such as the Gini index, and IM rates. Additionally, regarding the availability of professionals in PHC, Russo et al.^24^ found that an increase of one primary care physician per 10,000 inhabitants was associated with 7.08 fewer infant deaths per 10,000 live births. This suggests that, beyond other determinants, the presence of professionals adequately aligned with population size may be crucial in reducing IM rates.

Considering that the study was conducted during the years affected by the COVID-19 pandemic, it is essential to acknowledge its impact on MM in Brazil, particularly in the period following the implementation of PB. In 2021, the number of maternal deaths doubled when compared with 2019, highlighting the severe consequences of the pandemic on maternal health. Data indicate that from March 2020 onward, there was a 33.37% increase in the MM ratio, reflecting the heightened risks faced by pregnant women during this period. Although mortality trends stabilized in the subsequent months, this initial surge underscores the urgent need for robust maternal healthcare policies and interventions during public health crises^25^.

In this regard, it is important to note that federal funding programs are constantly evolving to meet demands and challenges within the Brazilian health system. One example is the new funding model for Maternal and Child Health, *Rede Alyne* (Ordinance GM/MS No. 5,350, September 12, 2024)^26^, which differs from PB. A key feature of *Rede Alyne* is the increase in financial transfers per pregnant woman, aimed at supporting states and municipalities in conducting prenatal exams. Although such funding mechanisms have contributed to higher prenatal care rates, they have not directly impacted maternal and infant mortality rates. This highlights the complexity of the issue and suggests that financial incentives alone may be insufficient. Therefore, future research could explore the impact of different funding models on improving maternal and child health outcomes across Brazilian municipalities.

This nationwide analytical epidemiological study has certain limitations that merit consideration. Firstly, the use of secondary data from sources such as Datasus, SIM, Sinasc, and Sisab may imply potential limitations related to the quality and reliability of the data. The validity of the findings depends on the accuracy and completeness of these databases; underreporting or inconsistencies may influence the conclusions of the study. Additionally, the use of aggregated data by municipality may obscure intra-urban variations that could be relevant for understanding the relationships between the studied variables.

Another limitation concerns focusing on quantitative variables, especially in the use of prenatal care rate as a key indicator, disregarding qualitative aspects of maternal and child healthcare. An exclusive emphasis on quantitative goals—such as the proportion of pregnant women attending prenatal consultations—may not fully reflect the quality of care provided, overlooking important nuances that can impact mortality outcomes. Finally, specific state or municipal policies and programs tailored to local needs may have contributed in reducing MM and IM rates. These programs, which were not included in the analysis, may have provided additional support or interventions that contributed to improved outcomes.

While these limitations do not invalidate our findings, they should be taken into consideration when interpreting and generalizing the results. Future research should address these gaps by incorporating longitudinal approaches, conducting more detailed assessment of data quality, and considering multiple determinants for a more holistic understanding of the complex dynamics associated with MM and IM.

## CONCLUSION

The new financing model introduced by PB led to an increase in prenatal consultations in Brazilian municipalities. However, it did not demonstrate a significant association with reductions in MM and IM between 2019 and 2022.

## Data Availability

The data are avalaible at: https://osf.io/bq6w2/DOI:10.17605/OSF.IO/BQ6W2.
